# Investigating the neural mechanisms of transcranial direct current stimulation effects on human cognition: current issues and potential solutions

**DOI:** 10.3389/fnins.2024.1389651

**Published:** 2024-06-18

**Authors:** Marcus Meinzer, Alireza Shahbabaie, Daria Antonenko, Felix Blankenburg, Rico Fischer, Gesa Hartwigsen, Michael A. Nitsche, Shu-Chen Li, Axel Thielscher, Dagmar Timmann, Dagmar Waltemath, Mohamed Abdelmotaleb, Harun Kocataş, Leonardo M. Caisachana Guevara, Giorgi Batsikadze, Miro Grundei, Teresa Cunha, Dayana Hayek, Sabrina Turker, Frederik Schlitt, Yiquan Shi, Asad Khan, Michael Burke, Steffen Riemann, Filip Niemann, Agnes Flöel

**Affiliations:** ^1^Department of Neurology, University Medicine Greifswald, Greifswald, Germany; ^2^Neurocomputation and Neuroimaging Unit, Department of Education and Psychology, Freie Universität Berlin, Berlin, Germany; ^3^Department of Psychology, University of Greifswald, Greifswald, Germany; ^4^Max Planck Institute for Human Cognitive and Brain Sciences, Leipzig, Germany; ^5^Wilhelm Wundt Institute for Psychology, Leipzig University, Leipzig, Germany; ^6^Department of Psychology and Neurosciences, Leibniz Research Centre for Working Environment and Human Factors at TU Dortmund, Dortmund, Germany; ^7^German Center for Mental Health (DZPG), Bochum, Germany; ^8^Bielefeld University, University Hospital OWL, Protestant Hospital of Bethel Foundation, University Clinic of Psychiatry and Psychotherapy, Bielefeld, Germany; ^9^Chair of Lifespan Developmental Neuroscience, Faculty of Psychology, Technische Universität Dresden, Dresden, Germany; ^10^Section for Magnetic Resonance, Department of Health Technology, Technical University of Denmark, Kongens Lyngby, Denmark; ^11^Danish Research Centre for Magnetic Resonance, Centre for Functional and Diagnostic Imaging and Research, Copenhagen University Hospital Amager and Hvidovre, Copenhagen, Denmark; ^12^Department of Neurology and Center for Translational Neuro- and Behavioral Sciences (C-TNBS), Essen University Hospital, University of Duisburg-Essen, Essen, Germany; ^13^Core Unit Data Integration Center, University Medicine Greifswald, Greifswald, Germany; ^14^German Center for Neurodegenerative Diseases (DZNE Site Greifswald), Greifswald, Germany

**Keywords:** tES, tDCS-fMRI, cognition, variability, experimental control, lifespan, design optimization, consortia

## Abstract

Transcranial direct current stimulation (tDCS) has been studied extensively for its potential to enhance human cognitive functions in healthy individuals and to treat cognitive impairment in various clinical populations. However, little is known about how tDCS modulates the neural networks supporting cognition and the complex interplay with mediating factors that may explain the frequently observed variability of stimulation effects within and between studies. Moreover, research in this field has been characterized by substantial methodological variability, frequent lack of rigorous experimental control and small sample sizes, thereby limiting the generalizability of findings and translational potential of tDCS. The present manuscript aims to delineate how these important issues can be addressed within a neuroimaging context, to reveal the neural underpinnings, predictors and mediators of tDCS-induced behavioral modulation. We will focus on functional magnetic resonance imaging (fMRI), because it allows the investigation of tDCS effects with excellent spatial precision and sufficient temporal resolution across the entire brain. Moreover, high resolution structural imaging data can be acquired for precise localization of stimulation effects, verification of electrode positions on the scalp and realistic current modeling based on individual head and brain anatomy. However, the general principles outlined in this review will also be applicable to other imaging modalities. Following an introduction to the overall state-of-the-art in this field, we will discuss in more detail the underlying causes of variability in previous tDCS studies. Moreover, we will elaborate on design considerations for tDCS-fMRI studies, optimization of tDCS and imaging protocols and how to assure high-level experimental control. Two additional sections address the pressing need for more systematic investigation of tDCS effects across the healthy human lifespan and implications for tDCS studies in age-associated disease, and potential benefits of establishing large-scale, multidisciplinary consortia for more coordinated tDCS research in the future. We hope that this review will contribute to more coordinated, methodologically sound, transparent and reproducible research in this field. Ultimately, our aim is to facilitate a better understanding of the underlying mechanisms by which tDCS modulates human cognitive functions and more effective and individually tailored translational and clinical applications of this technique in the future.

## 1 Introduction

Non-invasive brain stimulation (NIBS) techniques apply electric fields to the brain using currents injected via scalp electrodes (tES, transcranial electric simulation) or electromagnetic induction (TMS, transcranial magnetic stimulation). They aim to modulate the excitability of the human brain and induce neuroplasticity. NIBS has been ascribed great promise for allowing targeted modulation of specific brain regions or large-scale brain networks relevant for higher cognitive functions. In particular, transcranial direct current stimulation (tDCS) has recently sparked considerable scientific, clinical, and public interest (Dubljević et al., 2014; Riggall et al., 2015). Compared to other types of NIBS (e.g., transcranial magnetic stimulation, TMS), tDCS is relatively low-cost and easy to administer, has no significant adverse effects, and offers a relatively effective mode for placebo (sham) stimulation (Antal et al., 2017a).

The underlying neurophysiological mechanisms of tDCS have been studied extensively in the motor system by utilizing neuropharmacological interventions and TMS, to explore the modulation of cortico-cortical and cortico-spinal excitability (Cirillo et al., 2017). These studies have revealed that the applied current does not induce action potentials. Rather, it is suggested that tDCS transiently shifts the neuronal resting membrane potential toward either de- or hyperpolarization, resulting in enhanced or reduced neural excitability at the macroscale level with standard protocols (Sandrini et al., 2011; Stagg et al., 2018). Moreover, with regard to tDCS effects involving synaptic plasticity, animal and human studies have indicated that tDCS also introduces a secondary mechanism (in addition to alterations of the resting-membrane potential) that involves the induction of long-term potentiation and depression (LTP and LTD)-like processes (Stagg et al., 2018). Repeated stimulation sessions can enhance training-induced adaptive neuroplasticity and induce long-lasting behavioral improvements (Allman et al., 2016; Perceval et al., 2020).

TDCS has also been studied extensively with regard to its potential to enhance cognitive functions in healthy individuals and to treat cognitive impairment in various neurological and psychiatric diseases. However, recent reviews have noted substantial methodological variability, frequent lack of rigorous experimental control and overall, highly variable outcomes within and between studies (Galli et al., 2019; Lee et al., 2021; Lavezzi et al., 2022). Moreover, several recent registered reports have reported weak effects of tDCS, limited intra-individual reliability of tDCS responses or failed to replicate previous studies (Boayue et al., 2020; Alexandersen et al., 2022; Willmot et al., 2024). Nonetheless, a substantial body of research has demonstrated potential positive effects of tDCS on behavior and brain function, but the aforementioned issues also suggest that the current “state-of-the-art” may not yet be suited for translational applications. This would require systematic and coordinated evaluation of the parameter space, clarification of the underlying neural mechanisms, and likely individual adaptation of interventions based on this knowledge. Here, we review and critically discuss recent efforts that aim to address these important issues to improve the effectiveness of tDCS in experimental and clinical settings, as well as transparency and reproducibility of research outcomes in this field. We also aim to provide recommendations for future research investigating the neural mechanisms underlying tDCS effects on higher cognitive functions using neuroimaging technology (see Table 1 for a summary of the recommendations).

**Table 1:** Summary of key recommendations for imaging cognitive tDCS effects.

Planning stage	**Technologically challenging tDCS-imaging studies require careful planning** Assemble necessary expertise from relevant fields (e.g., physics, imaging, tDCS, data analysis and management) Consult relevant technical guidelines for tDCS and combined tDCS-imaging Address specific issues arising from combining tDCS with specific imaging approaches (e.g., safety, artifacts, distortions)
Imaging and task paradigms	**Consider the specific strengths and limitations of specific imaging approaches to answer the research question** e.g., spatial vs. temporal resolution, is structural MRI required (modeling) **Ensure compatibility of planned behavioral tasks with the imaging approach** Consider that task modifications may be required by specific imaging techniques and that changing behavioral tasks can affect tDCS effects Use robust and simple (few conditions) designs; maximize trial numbers Establish test–retest reliability of (adapted) designs Establish behavioral stimulation effects for (adapted) designs in the target population of the planned imaging study
tDCS	**Targeting** Neuronavigated targeting is preferred over scalp-based approaches, especially for focal set-ups Implement methods to minimize electrode displacement and verification of positioning accuracy relative to intended target regions (see Figure 1) **Stimulation** Individually optimized tDCS are preferable over uniform approaches in contexts that aim to maximize effectiveness Optimization can be enhanced by considering multiple sources (e.g., anatomy, modeling, fMRI) Focal tDCS is recommended for establishing causal brain-behavior relationships; conventional tDCS may have advantages in specific contexts (e.g., clinical populations)
Control	**Blinding** Triple blinding and use of optimized methods for participant blinding and assessment are recommended Reporting of blinding success and adverse effects is essential **Experimental design** Carefully consider the required level of experimental control (e.g., task, regional, timing, polarity or a combination of them) to answer specific research questions **Meta-control** Pre-registered reports are the best option to reduce bias and to enhance transparency (Note: early planning for protocol development and peer review is required) Pre-register methods, hypotheses and analytical approaches and clear statement of exploratory analyses are minimum requirements Implement open science principles, including FAIRification Adhere to relevant field specific (i.e., imaging, tDCS) guidelines for data analysis, sharing and reporting

## 2 Functional imaging to study effects of tDCS on higher-order human brain functions

Approximately 80% of the published tDCS studies target the primary motor cortex (M1), and it is currently unclear if results from these studies generalize to other cortical regions and brain networks, in particular those enabling higher cognitive functions (Stagg et al., 2018). Moreover, while neurophysiological effects of tDCS on local cortical excitability in the motor system can be assessed directly via modulation of TMS-induced motor evoked potentials (MEPs), this approach cannot be used to quantify neural effects of tDCS on cognition. Contemporary systems neuroscience research has also highlighted that cognitive functions are enabled by large-scale functional brain networks relying on coordinated processing across various regions (Breakspear, 2017; Pang et al., 2023). To date, however, there is still relatively little knowledge about how tDCS impacts these complex human brain networks, which can be addressed by combining tDCS with modern brain imaging techniques.

In principle, tDCS can be combined with different functional imaging techniques, including electroencephalography (EEG, Polanía et al., 2011; Okazaki et al., 2023), magnetoencephalography (MEG, Jang et al., 2017; Matsushita et al., 2021), functional near-infrared spectroscopy (fNIRS, McKendrick et al., 2015; Dutta, 2021) or functional magnetic resonance imaging (fMRI, Esmaeilpour et al., 2020; Jamil et al., 2020). However, combining tDCS with imaging technology requires careful consideration of appropriate designs and also decisions about which approach is best suited to answer specific research questions.

For example, imaging can be conducted either sequentially (before and after tDCS) or concurrently with tDCS. Sequential imaging allows the investigation of potential after-effects of the stimulation on brain function (Keeser et al., 2011; Shahbabaie et al., 2018) or can be used to interrogate the neural consequences of behavioral add-on effects when tDCS is administered during multiday training sessions or therapeutic interventions (Allman et al., 2016; Antonenko et al., 2023). In contrast, concurrent imaging and tDCS allows investigation of immediate tDCS effects on brain function (Esmaeilpour et al., 2020).

With regard to imaging modalities, EEG and MEG allow mapping of brain dynamics with excellent temporal resolution, which renders them optimal for investigating modulation of fast neural oscillations (Rossi et al., 2022). MEG and fNIRS are most sensitive to modulation of cortical regions (Attal et al., 2012; Li et al., 2023), and therefore less suited to investigate potential subcortical stimulation effects. In this review, we will focus on fMRI, because it allows investigating tDCS effects on brain dynamics with high spatial precision and sufficient temporal resolution across the entire brain. In addition, high resolution structural imaging data can be acquired in the same imaging session, allowing precise localization of potential stimulation effects, verification of correct electrode positions on the scalp and realistic current modeling based on individual head and brain anatomy (Hunold et al., 2023). These advantages have turned fMRI into the most widely used imaging technique to investigate the neural mechanisms underlying tDCS (Esmaeilpour et al., 2020), including pioneering intrascanner work that demonstrated acute modulation of ongoing brain activity at the stimulation site and large-scale neural networks (Meinzer et al., 2013; Antonenko et al., 2017; Jamil et al., 2020).

## 3 Investigating the underlying causes of variability in tDCS-fMRI studies

Since the reintroduction of tDCS at the turn of the 20th century (Priori et al., 1998; Nitsche and Paulus, 2000), numerous publications have reported promising effects of tDCS on motor and cognitive functions in health and disease, but also substantial intra- and interindividual variability of stimulation effects. This has prompted increased interest in investigating the underlying sources of variable tDCS responses, that are thought to be multifactorial and can broadly be classified as participant- and stimulation-dependent factors (Fertonani and Miniussi, 2017). In addition, developing optimal designs for either tDCS and fMRI studies can be challenging in itself. This is further complicated when both techniques are combined and design optimization may require creative solutions and specialist input from different fields. This will be discussed in the following sections.

### 3.1 Participant- and stimulation-dependent factors

Participant-dependent factors include trait- and state-dependent characteristics of the participants, including baseline behavioral performance, microstructural, metabolic and functional brain network variations between participants or intraindividual differences in intrinsic brain states of each participant at different stimulation sessions (Hordacre et al., 2017; Aberra et al., 2018; Antonenko et al., 2019a). The importance to account for these factors has been emphasized by studies showing that the tDCS response can be associated with demographic, behavioral, or neurofunctional characteristics of participants, including sex, age, education levels, genetics, cultural background, baseline task performance and neural network organization (Kuo et al., 2006; Berryhill and Jones, 2012; Martin et al., 2017b, 2019b; Antonenko et al., 2018; Fridriksson et al., 2019; Perceval et al., 2020; Ghasemian-Shirvan et al., 2022). Moreover, electric field modeling studies that considered individual head and brain anatomy have demonstrated associations between regional electric field strength and modulation of behavior, neurophysiological parameters, fMRI-derived brain networks, regional cerebral blood flow and neurochemical parameters (Kim et al., 2014; Cabral-Calderin et al., 2016; Antonenko et al., 2017, 2023; Jamil et al., 2020). These studies have highlighted the contribution of various participant characteristics to variable tDCS responses that require more systematic investigation in the future.

On the other hand, many stimulation-dependent factors, like the timing, intensity or duration of tDCS are determined by a given experiment. Yet, even minor modifications to experimental protocols can alter the outcomes (e.g., Gauvin et al., 2017), thereby contributing to differences between studies. However, many of these factors can also interact directly with participant characteristics. For example, while the intensity of the induced current was held constant in the majority of previous tDCS studies, it has been convincingly suggested that individual skull (Datta et al., 2009; Bikson et al., 2012; Hanley and Tales, 2019; Sun et al., 2021), or brain anatomy (Suh et al., 2012; Dahnke et al., 2013; Filmer et al., 2019), critically determine how much current reaches the target regions for tDCS, resulting in variable current dose in the target regions. Moreover, accurate positioning of the electrodes on the participants’ scalp, one of the most critical stimulation dependent factors, can be affected by experimenter error (i.e., electrode misplacement) or incremental drift over the course of the experiment, resulting in current flow variations between participants (Woods et al., 2016; Indahlastari et al., 2023). This issue might be even more relevant for focal tDCS set-ups, that constrain the current flow to circumscribed brain regions (Villamar et al., 2013; Gbadeyan et al., 2016b), because the regional specificity of the administered current renders these setups particularly vulnerable for deviations from intended electrode positions, resulting in reduced current dose in target regions for tDCS (Niemann et al., 2024).

**Figure 1:**
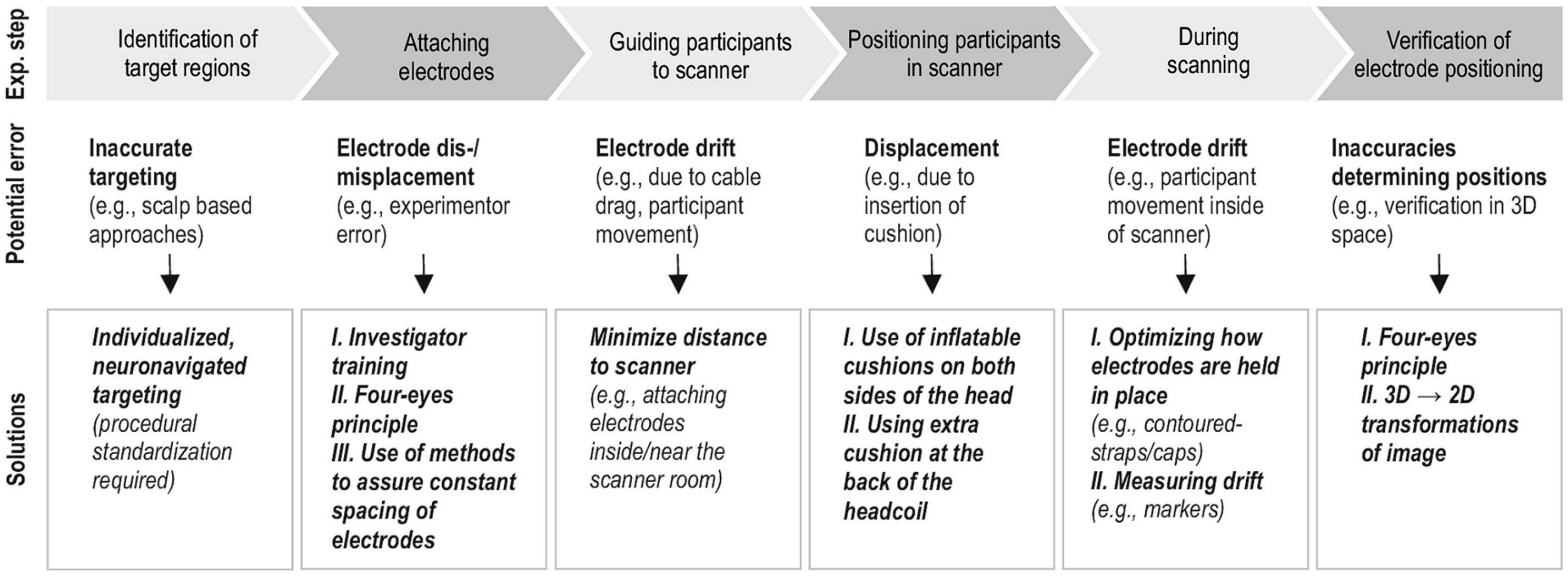
Illustrates factors that can affect electrode position accuracy during concurrent fMRI-tDCS experiments and subsequent verification. Suggestions are based on our own experience during an ongoing multicenter, intrascanner tDCS-fMRI project (www.memoslap.de/). The upper panel shows the different phases of a concurrent tDCS-fMRI study and subsequent identification of electrode positions based on structural images of individual participants. Below, examples for potential sources of errors (middle panel) and possible solutions (bottom panel) are described. An example for a method to assure constant spacing of electrodes for a 3 × 1 focal montage that uses a 3D printed spacer can be found in Niemann et al. (2024). Because verification of electrode positions based on 3D structural images can be challenging, transformation into a 2D “pancake view” of the reconstructed scalp may be suited to facilitate identification of actual scalp positions of tDCS electrodes (De Munck et al., 2013; Fleury et al., 2019). Fully automated methods based on neural network models are currently being developed by our group.

Of note, complex intrascanner tDCS-fMRI studies that require participants to walk to and be positioned inside the scanner with electrodes attached, are at high risk for electrode displacement and this effect may vary depending on target sites (e.g., electrodes positioned underneath the cushions that are used to stabilize the head may be more likely to move). Specific problems pertaining to mis- and displacement of electrodes during tDCS-fMRI studies and subsequent verification of correct electrode positions are illustrated in Figure 1, along with suggestions how to minimize them. Therefore, future research in this field should routinely implement appropriate methods not only for improving electrode positioning prior to scanning (e.g., electrode placement guided by neuronavigation) (De Witte et al., 2018), but also implement methods to minimize electrode displacement and drift, verify electrode positions before and/or after functional imaging, and consider empirically determined actual electrode positions when dose–response relationships are investigated (Woods et al., 2015; Knotkova et al., 2019; Antonenko et al., 2019b; Albizu et al., 2023; Niemann et al., 2024).

### 3.2 Design considerations for tDCS-fMRI studies

Investigating and controlling for variability in tDCS-fMRI studies can be challenging because stimulation effects are not assessed by a direct marker of brain physiology (i.e., MEPs), but rely on proxy measures of brain function (i.e., variable behavioral performance in task-related fMRI and indirect measures of neural activity, like the blood oxygenation-dependent response). Behavioral performance parameters can be significantly influenced by numerous internal and external factors. For example, improvements in performance across repeated sessions have been linked to familiarization and training effects, or the development of cognitive strategies (Bell et al., 2018). These confounds are particularly relevant for cross-over designs frequently used in tDCS-fMRI studies and can result in reduced effect sizes, even when the stimulation conditions are appropriately balanced across participants (Falleti et al., 2006; Hausknecht et al., 2007; Bartels et al., 2010). While these confounds can be mitigated to some degree by choosing robust designs and implementation of parallel task versions, it is advisable to formally establish test–retest reliability of experimental paradigms in the specific target populations (e.g., young vs. older individuals, clinical populations of interest) prior to implementation in costly tDCS-fMRI studies and to consider the outcomes when interpreting effect sizes of behavioral and neural modulation.

Moreover, specific paradigms or populations are particularly challenging for tDCS-fMRI studies. For example, learning paradigms are typically associated with performance increases across time, therefore the number of correct responses and errors vary depending on learning stage. Inclusion of both response types in the analysis can be problematic, because the neural signatures differ (Postman-Caucheteux et al., 2010). Moreover, restricting the analysis to correct trials reduces statistical power to detect learning specific neural activation in early stages or in different tDCS conditions. One possible solution for this problem was suggested by Sliwinska et al. (2017), who used an associative picture-word learning paradigm and provided feedback about correct associations after each trial. This feedback-based design assured that “learning” was possible even following incorrect responses, thereby allowing to investigate a common neural process across all trials. Moreover, behavioral responses can be highly variable *per se* in certain populations, which can mask potential neural tDCS effects. This was addressed by Darkow and Flöel (2016), who investigated tDCS effects during a picture naming task in patients with chronic language impairment (aphasia). In this study, only object pictures that could be named consistently by the patients across several baseline naming assessments were used. This maximized the number of correct responses during a subsequent cross-over tDCS-fMRI study and allowed imaging of the neural effects of tDCS on residual language networks, independent of performance.

Another important aspect specific to imaging of tDCS effects pertains to the robustness of the imaging procedure itself, that can be affected by a number of different factors including physiological noise due to cardiac and respiratory cycles, head motion artifacts, magnetic field inhomogeneities, and fMRI signal drift. These factors can contribute to variability in cross-over and longitudinal tDCS-fMRI studies and need to be monitored and considered in data analysis (Esmaeilpour et al., 2020). However, certain aspects of tDCS-fMRI studies can also be addressed at the design level. For example, fMRI signal drift depends on gradual heating of the MRI scanner and can be controlled for to some extent by scanning participants at the same time of day or by “warming up” the scanner prior to each session. However, this does not address within-session effects, i.e., signal changes from the early to later stages of a paradigm. An elegant solution to this problem was suggested by Sliwinska et al. (2017) in their picture-word association learning paradigm. The authors grouped learning trials in a way that different stages of the learning process (i.e., low vs. higher proficiency) were achieved repeatedly across consecutive micro-blocks, thereby minimizing the effect of signal drift on fMRI activity for each learning stage. Notably, numerous other factors have been shown to reduce reliability in task-related imaging protocols, including participant characteristics (e.g., advanced age, clinical populations > young healthy participants), design (e.g., long > short retest intervals; event-related > block designs) or specifics of data analysis (e.g., univariate or region-of-interest > multivariate analyses, complex > simple contrasts; for review see Noble et al., 2021). Because these factors can also increase variability in tDCS-fMRI studies, they require careful consideration at the design stage, to minimize the risk of masking potential tDCS effects on behavior and brain functions.

Additional consideration pertains to imaging artifacts that are induced by conventional and focal set-ups. While image distortions are typically limited to the scalp and skull, signal-to-noise (SNR) reductions in the functional images, that are most pronounced underneath the location of the electrodes, have been reported (Saiote et al., 2013). SNR reductions are most pronounced for the comparison between images acquired with and without electrodes (Gbadeyan et al., 2016b), but occur to a lesser degree also for the comparison of active vs. sham conditions and may vary between brain regions (Antal et al., 2011; Holland et al., 2011). Therefore, careful quantification of potential imaging artifacts and SNR reductions associated with specific equipment and target regions is necessary, and the outcomes should be considered in the design of the study. For example, when effects of different active stimulation sites are of interest, the between site effect needs to be controlled by comparison with its own sham condition with a similar regional SNR profile.

In sum, investigating the variability underlying tDCS effects using fMRI requires careful consideration of generic issues relevant for each individual approach (e.g., robustness of designs, accurate placement of electrodes), but also those that are specific to their combined use (e.g., imaging artifacts induced by the tDCS set-up, drift of electrodes during imaging, design optimization for imaging and tDCS), which can be challenging. However, tDCS-fMRI approaches also offer unique opportunities to tease apart the contribution of participant-, stimulation- and design-dependent factors on the variability of tDCS effects (e.g., structural MRI allows to estimate effects of anatomical variability on current flow patterns; comparison of tDCS administered during task performance or rest investigate neural stimulation effects on a constrained vs. unconstrained set of brain regions), thereby contributing to the development of future individualized tDCS approaches with potential to enhance the effectiveness of this technique in experimental and translational human neuroscience.

## 4 Optimization of tDCS protocols

The specific montages used to administer tDCS (e.g., conventional vs. focal set-ups, size and positioning of electrodes) affect the intensity and distribution of the induced current. Conventional set-ups use relatively large electrodes (e.g., 5 × 7 cm, up to 10 × 10 cm) that are typically attached over cortical regions in different hemispheres and induce a relatively wide-spread current flow affecting multiple neural networks. This lack of focality renders them less desirable for revealing regionally specific, causal brain-behavior relationships compared to focal montages. Those use smaller and often concentrically arranged electrodes in the same hemisphere to constrain the current to circumscribed brain regions (Kuo et al., 2013; Bortoletto et al., 2016). Notably, conventional setups may be more resilient to positioning errors and electrode drift compared to focal set-ups and electrode displacement has been shown to result in physiologically significant reductions in current dosage specifically within the immediate target regions (Niemann et al., 2024). This is particularly relevant for tDCS-fMRI studies, that are at high risk for electrode displacement, e.g., due to positioning of participants in the scanner after electrode attachment (see discussion above). Conventional set-ups may also have advantages in contexts where experimenter error is more likely to occur (e.g., routine clinical care, multicenter intervention studies) or specific clinical populations with variable lesion patterns and functional reorganization (Darkow et al., 2017). Therefore, the choice of montage in tDCS-fMRI studies strongly depends on the specific research question and population.

Furthermore, the majority of tDCS research has relied on the 10–20 (or 10–10) EEG system to identify target brain regions (Thair et al., 2017). This approach involves manual or automatic identification of anatomical markers (e.g., nasion, inion, preauricular points) and additional measurements (e.g., head circumference, calculation of intersections between landmarks) to determine the intended scalp positions of electrodes. Depending on which system is used, 25 or 74 reference points are available and placement is often guided by electrode caps (Tsuzuki et al., 2016). While this approach considers head size of individual participants to some degree, other properties of brain and skull morphology are neglected, resulting in a loss of precision (Herwig et al., 2003; De Witte et al., 2018). Structural MRI-guided neuro-navigation is a more individualized localization technique, which has mainly been used in experimental and clinical TMS studies, but more recently also for positioning of tDCS electrodes (De Witte et al., 2018; Lioumis and Rosanova, 2022). This approach requires a high-resolution structural T1-weighted image of individual participants that can be acquired prior to a tDCS-fMRI study. By co-registration of the structural image with a three-dimensional brain model and use of specific soft- and hardware for identifying the target brain regions, electrode positioning accuracy relative to individual brain anatomy can be improved (De Witte et al., 2018). Because of significant variations in brain anatomy and head shape, individualized neuronavigation-based electrode placement is currently the best option to improve positioning of electrodes at the intended scalp positions in tDCS-fMRI studies and scalp-based approaches are discouraged, especially for focal set-ups.

Individual differences in brain anatomy are crucial not only for precise placement of electrodes but also for optimizing the distribution of tDCS-induced electric fields within individual brains (Kim et al., 2014; Bikson et al., 2015; Antonenko et al., 2021). Previous studies have also highlighted the importance of investigating fundamental aspects of the induced electric field, such as current strength, focality, and its dependency on anatomical features of the head (Edwards et al., 2013; Opitz et al., 2015; Saturnino et al., 2015). In this context, computational models are frequently used to estimate the strength of the cortical electric field, since direct measurement in the human brain is not feasible except in highly selected patient populations (e.g., tumor resection, brain surgery for epilepsy treatment; e.g., Huang et al., 2017).

Recently, individualized electric field calculations have allowed investigating correlations between the individually received physical stimulation dose and the physiological impact of tDCS (for review see Hunold et al., 2023), known as cortical dose–response relationship. Moreover, manufacturers of brain stimulation devices are increasingly interested in updating their devices’ capabilities to estimate the electric field via computer modeling techniques, which rely on electrode positioning and stimulation current intensity. For instance, Soterix HD-Explore^1^ is a commercial, stand-alone software that models the current flow using the finite element method to estimate the electric field distribution for complex tDCS set-ups. However, it is essential to note that computer simulations estimate the electric field based on assumptions about electrical conductivity of different tissue classes and that they depend on the anatomical accuracy of the (semi-)automatic tissue segmentations obtained from the MR images. Therefore, validation of these assumptions and assessment of the segmentation accuracy is critical to improve the accuracy of computational models, and simulation errors may obscure potential associations between estimated fields and the recorded behavioral or neural response. In this context, Magnetic Resonance Current Density Imaging (MRCDI) and Magnetic Resonance Electrical Impedance Tomography (MREIT) are emerging techniques to investigate tDCS-induced current flow conductivity of brain tissue during concurrent tDCS-MRI measurements, respectively (Göksu et al., 2018, 2019). These modalities have potential for validating tDCS electric field simulations and optimizing individualized current dose calculations in both healthy participants and patients.

**Figure 2:**
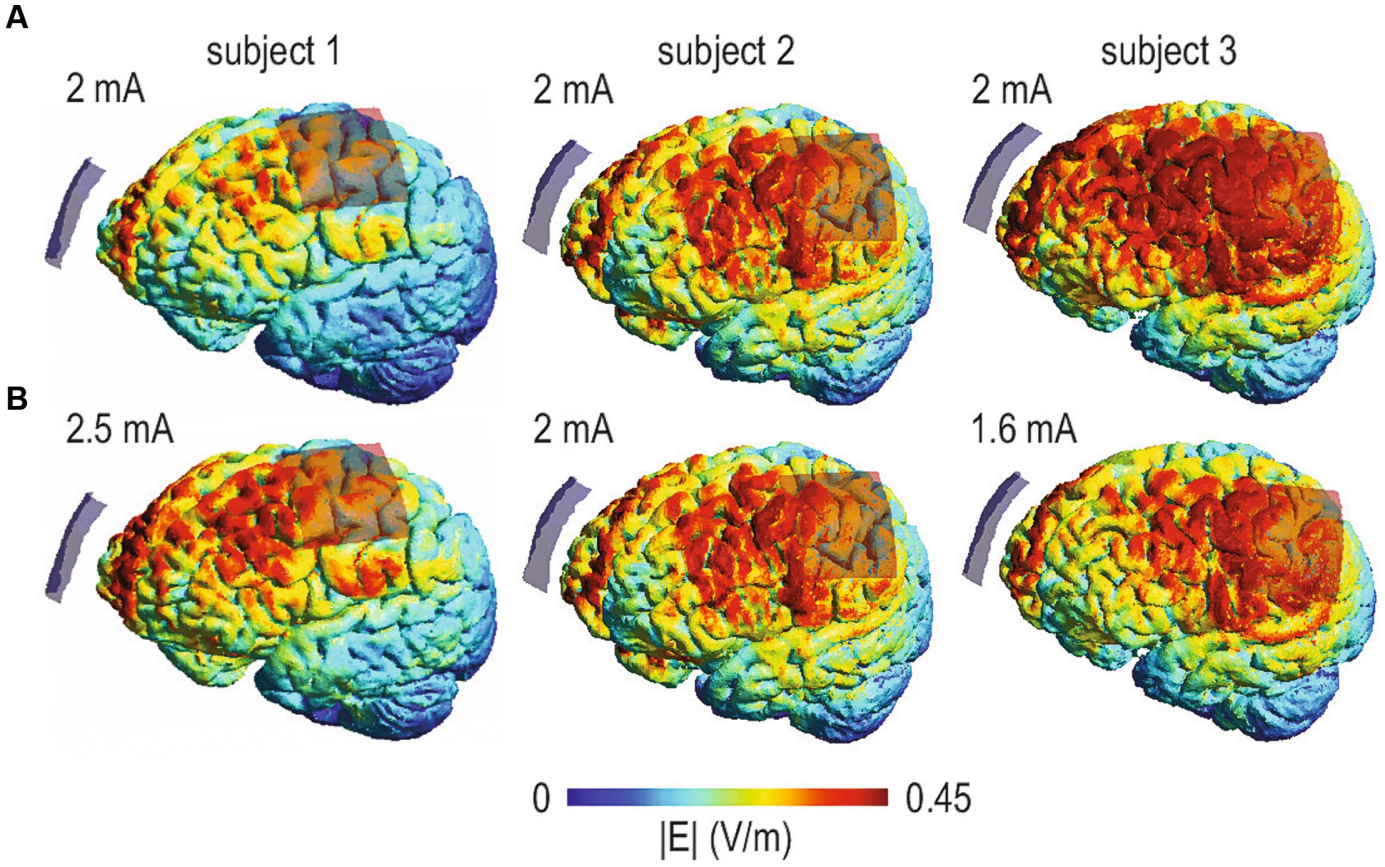
Computational models of tDCS-induced electrical current flow based on structural MRI data of three participants and a conventional montage targeting the left primary motor cortex (anode). Cathodes are positioned over the contralateral supraorbital region. Colors illustrate the distribution and intensity of the current (electric field strength, V/m). Only the left side of the brain is shown. **(A)** When individual head and brain anatomy is considered in computational models of current flow, the same intensity (i.e., tDCS administered with 2 mA) can result in highly variable current flow in individual participants. **(B)** Provides a simple example of dose control. Simulations were conducted with different intensities (i.e., 2.6, 2.0, and 1.5 mA) to minimize differences between participants.

Another issue that has recently gained substantial attention in tDCS studies is dose control. In this context it is important to note that even when the same current intensity is applied, participant-specific factors like skull and brain anatomy and others are major determinants of the actual current dose arriving in the target regions (Evans et al., 2020; Antonenko et al., 2021). This is illustrated in Figure 2. Hence, recent studies have used computational current modeling approaches to optimize stimulation intensity across participants. For example, Caulfield et al. (2020) acquired structural MRI data of individual participants to compute a “reverse-calculated tDCS dose” of tDCS applied at the scalp required to induce a uniform E-field (arbitrarily set at 1.00 V/m and not yet empirically tested in a prospective stimulation study) in a region-of-interest in the primary motor cortex. Notably, the minimum current intensity threshold for physiological modulation in different brain regions is unknown and may vary between stimulation sites and individuals. Moreover, increasing intensity does not necessarily increase neurophysiological effects and even changes in polarity have been observed. For example, while cathodal tDCS with 1 mA and 3 mA induced inhibitory effects, 2 mA may result in enhanced excitability (Batsikadze et al., 2013; Mosayebi Samani et al., 2019).

Therefore, currently little is known about objective optimization criteria for field intensity (Lee et al., 2021). Moreover, reaching a pre-determined criterion may require intensities beyond accepted safety thresholds in individual participants (Antal et al., 2017a) and in the study above, the required current intensity across participants ranged from 3.75–9.74 mA (Caulfield et al., 2020). Therefore, alternative approaches are currently being developed that use a combination of individual electric field modeling and anatomical information to enhance regional precision (Rasmussen et al., 2021) or the development of prospective dosing strategies aimed at matching the average field dose in different target regions at the group level, while maintaining dose variability for each region to enable systematic tests of dose–response relationships (Saturnino et al., 2021). However, in order to be valuable, optimization approaches should be informed by multiple sources (e.g., underlying anatomy, computational estimation of field magnitude, focality or functional task-dependent activity for optimization of stimulation targets) rather than being unidimensional.

## 5 High-level control

Achieving high-level experimental control is crucial not only to ensure reliable and valid results, but also to establish causal relationships in tDCS studies. In this context, we will discuss two major aspects relevant to tDCS-fMRI studies: (a) blinding of participants and research staff and (b) design-related issues pertaining the establishment of valid assumptions about the relationship between brain stimulation and behavioral and neural modulation. A separate section, we will discuss “control” from a broader perspective, i.e., in the context of open science practices and recent efforts to increase the validity, reproducibility and transparency of empirical research, which is highly relevant for tDCS-fMRI studies.

### 5.1 Blinding

The majority of previous tDCS studies have relied on placebo (“sham”) tDCS conditions to control for placebo effects. Sham-tDCS typically involves gradually increasing the current to the target intensity (e.g., over 10 s), followed by an immediate or briefly delayed fade-out period, during which the current intensity is decreased to zero (Huey et al., 2007; Axelrod et al., 2015; Jaberzadeh et al., 2019). Sham-tDCS protocols intend to elicit a transient physical sensation on the scalp (e.g., tingling, itching) that closely resembles the sensation experienced during active stimulation, but without inducing physiologically relevant effects on brain function due to the short duration of the stimulation. Sham protocols have been suggested to allow for effective blinding of participants (Gandiga et al., 2006) without modulating corticospinal excitability, CSE (Dissanayaka et al., 2018). However, several recent studies have also questioned blinding efficacy of specific sham-tDCS protocols (O’Connell et al., 2012; Wallace et al., 2016; Greinacher et al., 2019; Turi et al., 2019). For instance, Greinacher et al. (2019) probed blinding integrity every 30 s during a low-intensity active or sham tDCS protocol (30 s ramp-up/down, 600 vs. 20 s active 1 mA M1-tDCS) and demonstrated that participants could identify active tDCS in approx. 60% of the probes with high confidence.

These findings have recently led to the development of novel sham protocols that minimize differences between active and sham conditions in tDCS studies. For example, Neri et al. (2020) introduced a new sham-tDCS approach for multi-electrode tDCS that used computational current modeling to optimize electrode positions during sham in a way that zero or very low magnitude electric fields are delivered to the brain, while medium to high intensity currents are maintained in at least some scalp electrodes. Notably, participant blinding for this new approach was superior compared a conventional bifocal montage and the desired blinding effect was achieved without eliciting a significant effect on CSE (Neri et al., 2020). These findings suggest that blinding efficacy reported for conventional sham protocols may need to be interpreted with caution (O’Connell et al., 2012) and alternative protocols that minimize differences between active and sham-tDCS may be more appropriate for achieving participant blinding.

In addition, rigorous staff blinding is crucial for preventing experimenter effects, such as the introduction of selection bias, observer bias, or inadvertent effects on experimental outcomes or during data analysis (Grimes and Schulz, 2002; Holman et al., 2015). Blinding of experimenters conducting the experiment and interacting with participants can be difficult with some commercially available stimulators (e.g., with indicator lights or sounds indicating on vs. off conditions), unless a two-experimenter approach is adopted: one administering the stimulation, while the second remains blinded while interacting with the participant (Reinhart et al., 2017). However, the majority of modern stimulators can now be equipped with advanced study modes, enabling easy customization for various stimulation conditions and parameters that can be triggered by pre-assigned codes. These new developments minimize the risk of unintentional unblinding of the experimenter and it is highly advisable to use such approaches in tDCS-fMRI studies. In addition, blinding of staff during data analysis is also advised and can be achieved by masking the stimulation conditions (i.e., by using participant codes that do not reveal active and control conditions).

The final issue pertains to how blinding is assessed. Here, a common practice involves a post-stimulation questionnaire, serving two key purposes: (a) directly valuating participants’ capacity to differentiate between stimulation conditions, which is frequently complemented by (b) self-reported assessment of potential side effects (e.g., tingling or burning, changes in mood or concentration levels) experienced during the stimulation (Ambrus et al., 2012; Turi et al., 2019). Although end-of-study questionnaires have been considered valid measures for evaluating the effectiveness of blinding (Antal et al., 2017b), a recent study by Turner et al. (2021) reported that the accuracy of end-of-study guesses was not more reliable than chance in predicting participants’ ability to distinguish between active or sham tDCS. Hence, it was suggested to incorporate additional online probe questions during the stimulation process for more accurate evaluation of blinding efficacy if possible. In any case, careful documentation of methods and results of participant and staff blinding is essential in all tDCS studies (Ekhtiari et al., 2022).

### 5.2 Establishing causality in tDCS studies

Another aspect particularly relevant for tDCS-fMRI studies pertains to establishing causal relationships between stimulation effects and behavioral and neural modulation. This cannot be achieved by comparing the effects of active vs. sham tDCS alone, because the latter only controls for potential placebo effects. In principle, stronger causal assumptions for the relevance of a given brain region to specific behavioral outcomes are potentially possible by additional direct comparison of anodal-excitation and cathodal-inhibition effects (AeCi). However, AeCi effects have rarely been demonstrated for cognitive tasks, mainly due to relatively weak or variable inhibitory effects of cathodal tDCS on cognition, which may be explained by redundancy within the neural networks supporting higher-order cognitive functions (Jacobson et al., 2012). Moreover, cathodal tDCS has also been shown to enhance performance during specific tasks, presumably by enhancing signal-to-noise during cognitive tasks (Antal et al., 2004).

However, several other approaches to achieve high-level experimental control in tDCS-fMRI studies are suitable and depend on the specific research question. For example, regional specificity of tDCS effects can be investigated by including one or more additional active control stimulation sites, specifically targeting cortical regions outside of the neural network(s) involved in processing of the task of interest. This approach not only allows to investigate unspecific (placebo) effects, but also the specificity of neural network modulation relative to the respective task. For example, Gbadeyan et al. (2016a) investigated behavioral effects of focal tDCS administered to either the left or right dorsolateral prefrontal cortex (dlPFC) and M1 during a visual Flanker task. Prefrontal active vs. sham tDCS improved adaptive cognitive control, thereby confirming involvement of both left and right dlPFC in this specific process. The absence of stimulation effects after left or right M1 tDCS demonstrated regional specificity. Notably, higher-order cognitive functions are often supported by multiple brain regions that are organized in partially overlapping neural networks. Therefore, selecting suitable and meaningful active control sites can be challenging.

Regional specificity can be complemented by the investigation of task specificity of tDCS effects. At the lowest level of control, the latter involves two or more different tasks, that are completed while the same cortical region is stimulated. This allows controlling for unspecific effects of the stimulation and demonstration that a given region or network is involved in task A, but not B. For instance, Martin et al. (2017a) demonstrated improved performance in a visual perspective taking task when focal anodal tDCS was administered over the dorsomedial frontal cortex (dmPFC). No significant change was observed in a source memory task with the same tDCS intervention, which illustrates a simple case of task-specificity of tDCS effects. Another highly specific aspect of task specificity pertains to activity selective stimulation effects (Bikson et al., 2013). This implies that even though several brain regions may be affected by the current, only those activated by a specific task are susceptible to the effects of tDCS. To the best of our knowledge, this assumption has not yet been tested with functional imaging. In principle, this could be investigated with a conventional montage that induces relevant current in neighboring brain areas that are differentially activated by two tasks. In this context, it would be predicted that the same montage preferentially modulates activity in the respective task-relevant regions and networks.

Moreover, one of the highest levels of experimental control at the design stage can be achieved by the combined investigation of regional and task specificity. This requires a minimum of two stimulation sites targeting processes or neural networks relevant for different tasks. For example, Martin et al. (2019a) investigated effects of focal tDCS administered to either the right TPJ or dmPFC on social cognition, including visual perspective taking tasks requiring line-of-sight and mental rotation judgments. Using this approach, the authors demonstrated a double dissociation of behavioral tDCS effects, indexed by specific facilitation of embodied mental rotation of the self into an alternate perspective by rTPJ tDCS, while dmPFC tDCS facilitated integration of social information relevant to self-directed processes.

Finally, analysis of specificity is not limited to tasks and regions, but also applies to the timing of the stimulation relative to a given task (temporal specificity). The majority of previous studies have employed single tasks and investigated behavioral effects of tDCS administered at different time points (i.e., prior to, during or after the task). These studies have highlighted that maximal tDCS effects may be achieved with varying timing across different functional domains, including visuomotor and visuospatial skills (Reis et al., 2015; Oldrati et al., 2018), motor network modulation (Calzolari et al., 2023) and language processing (Cao and Liu, 2018). Moreover, timing specific neurophysiological or behavioral modulation have been reported in different populations (i.e., young vs. older adults, for review see Perceval et al., 2016). Hence, these factors also need to be considered in the design phase of future tDCS-fMRI studies, e.g., by establishing optimal stimulation time windows in prior behavioral studies.

### 5.3 Scientific rigor and integrity beyond the experimental context

From a broader perspective, high-level control also includes the promotion of open and transparent research practices (Munro and Prendergast, 2019). For example, fMRI data analysis is a complex process that can be accomplished using a variety of platforms and analytical approaches that frequently comprise custom code. This was highlighted by Botvinik-Nezer et al. (2020), who demonstrated that of 70 labs that were asked to analyze the same fMRI dataset, all used different workflows. In the context of the ongoing replication crisis in science (Open Science Collaboration, 2015), appropriate documentation of data analysis procedures, code, and optimally pre-registration of analytical steps is highly desirable when investigating tDCS effects using imaging. This will facilitate the interpretation of the results and enhance the validity of research in this field (Esmaeilpour et al., 2020). Transparency, reproducibility and availability of data and analytical approaches can be further enhanced by adhering to relevant guidelines for data analysis and sharing (e.g., Gorgolewski and Poldrack, 2016; Nichols et al., 2017) and the FAIR (Findable, Accessible, Interoperable, Reusable) guiding principles for scientific data management and stewardship (Wilkinson et al., 2016).

Another important issue pertains to publication bias favoring positive outcomes, either because researchers do not attempt to disseminate negative results, or are discouraged by publishers to submit even valid negative findings. A particularly powerful approach to prevent publication bias are registered reports, where a study is accepted for publication based on its merit to answer a specific research question, irrespective of the eventual outcomes (that will be published alongside the protocol after study completion). Unlike pre-registration, the methodology and hypotheses of the planned study undergoes peer review, which helps to prevent publication of negative results based on methodological flaws (Chambers and Tzavella, 2021). Pre-registered reports are particularly relevant in the context of recent meta-analyses and systematic reviews of tDCS effects on human cognition (Galli et al., 2019; Yan et al., 2020; Figeys et al., 2021; Majdi et al., 2022), that have discussed not only limited reproducibility and small sample sizes in this field, but also the risk of p-hacking or HARKing (i.e., Hypothesizing After the Results are Known). Pre-registrated reports effectively address these biases and also provide a robust foundation for hypothesis-driven research and confirmatory replication. Notably, several hundred of journals now offer this option, including high profile neuroscience and neuroimaging outlets that are of interest for tDCS-fMRI studies (e.g., Nature Communications, NeuroImage, Cortex). Nevertheless, we would also like to emphasize the importance of data-driven and exploratory analyses. This particularly relevant in a relatively novel and evolving fields of science like tDCS-fMRI and the multitude of parameters that can influence stimulation success. In this context, clearly stated exploratory analyses can generate new hypotheses and serve as starting points for subsequent confirmatory studies.

## 6 Systematic assessment of tDCS effects across the human lifespan

Translational tDCS research that aims at counteracting age-associated decline or impairment of cognitive functions has yielded promising but mixed results so far (e.g., Perceval et al., 2016). For example, studies that directly compared tDCS effects in young and older adults have demonstrated larger behavioral effects in younger (Ross et al., 2011; Manenti et al., 2013), while others revealed larger effects in older adults (Zimerman et al., 2014; Cespón et al., 2017; Perceval et al., 2020). Moreover, while some studies have suggested that tDCS can improve (impaired) performance in older adults to the level of younger adults (Meinzer et al., 2013), others found detrimental effects in older adults when using the same montage that improved behavioral functions in young adults (Boggio et al., 2010; Fertonani et al., 2014). These findings are not surprising, because the neural substrates that support cognition and motor function in young and older adults can differ substantially and therefore, positive results obtained in tDCS studies involving young individuals cannot automatically be translated to older populations (Perceval et al., 2016). This needs to be investigated more systematically in the future across functional domains and the entire human lifespan.

Furthermore, neural aging, which for most cognitive domains becomes apparent by the end of the third decade of life (e.g., Hedden and Gabrieli, 2004), is not a uniform process and the degree of functional and structural brain reorganization is influenced by a number of intra-individual, environmental and life-style factors (Gutchess, 2014). In addition, age-related changes in brain structure, cerebrospinal fluid/brain ratio or skull thickness may affect the degree or distribution of the induced current itself (Opitz et al., 2015; Indahlastari et al., 2020; Antonenko et al., 2021). These aging-related factors may explain differences in stimulation response between young and older individuals, but also variability of stimulation effects within older population. To date, however, it has not yet been systematically investigated how potential differences in current flow due to brain atrophy or other factors, reported to significantly impact current flow based on simulation studies (Indahlastari et al., 2020), and interactions with age-associated functional network reorganization, affect behavioral and neural tDCS effects. TDCS-fMRI approaches are particularly suited to investigate these important issues, because they can provide information on both baseline neural network organization and functional changes due to the stimulation. Moreover, structural imaging data can be acquired in the same session and subsequently be used for individualized current modeling. Consideration of these variables in data analysis has great potential to reveal the underlying mechanisms and predictors of stimulation response across the human lifespan.

Finally, many neurological conditions (e.g., stroke-induced motor or cognitive impairment, dementia and precursors) primarily occur in elderly individuals, thereby a pathological process is superimposed on “normal” age-associated structural and functional brain reorganization. Hence, a better understanding of how these age-associated brain changes interact with tDCS also has direct implications for enhancing the clinical application of this technique in the future (Crosson et al., 2015).

## 7 Establishing large-scale consortia for coordination of tDCS research

Developing research consortia has yielded unprecedented insights and facilitated discovery research in many fields of basic and translational neuroscience (e.g., Human Brain Project^2^; ENIGMA^3^), by strengthening of research capacity through pooling of resources and expertise and generating standardized outcomes and solutions for a common set of questions (Tagoe et al., 2019).

To date, however, a significant proportion of tDCS studies have been limited by small sample sizes and highly variable methodological approaches (Minarik et al., 2016; Hiew et al., 2022). Given the vast parameter space of tDCS experiments (e.g., montage, current intensity, target region, polarity, control condition; Sergiou et al., 2020; Kurtin et al., 2021), there are rarely comparable studies with similar protocols, precluding definitive conclusions regarding the effects of tDCS on cognition or establishing optimal protocols for specific research questions. Consortia can effectively address these issues by facilitating participant recruitment from multiple contributors using coordinated methodology and outcome measures. The increased diversity of the sample can in turn increase the generalizability of research findings and concurrent recruitment of participants expedites data acquisition, thereby accelerating the research process. This is currently being addressed for the first time by a recently funded project in Germany, that employs highly coordinated tDCS-fMRI and computational approaches to systematically investigate the underlying neural mechanisms and predictors of tDCS effects on learning and memory across different functional domains and the human lifespan. This consortium will also play an important educational role by providing training opportunities for junior scientists and researchers through high-quality brain stimulation workshops and conferences, to foster knowledge exchange, skill development, and networking. Notably, a similar global approach has been initiated by the International Network of Neuroimaging Neuromodulation (INNN), a group comprising experts and early-mid career researchers, that conducts regular workshops and education seminars that are publicly available via a YouTube channel.^4^

Furthermore, consortia play a vital role in monitoring and standardizing the execution and reporting of tDCS interventions. Importantly, combining tDCS with fMRI requires specific equipment, poses multiple technological challenges (e.g., safety assurance and management of potential imaging artifacts introduced by the equipment). Consequently, research consortia should aim to bring together expertise not only from brain stimulation, but also invite collaborators from additional relevant fields with specific expertise in neurophysics, engineering, neuroimaging methodology and data analysis and also data management, to further strengthen this field. These approaches will be highly relevant, because development of large-scale coordinated tDCS-fMRI datasets will require advanced and automated analytical procedures like machine learning and other data-driven approaches. For example, machine learning algorithms have shown great promise in predicting tDCS response in small scale studies based on a variety of factors, including current intensity and direction (e.g., Albizu et al., 2020). Adapting and fine-tuning these methods for the use in large-scale samples will likely be the next frontier in increasing our understanding of the neural mechanisms and predictors of tDCS response.

Finally, the lack of sufficient methodological and procedural information frequently hinders reproducibility and further advances in this field (Esmaeilpour et al., 2020). This was recently addressed by an international consortium (The International Network of Transcranial Electrical Stimulation-fMRI, tES-fMRI) by establishing a consensus-based standard for reporting essential details in concurrent tES-fMRI studies (Ekhtiari et al., 2022). The checklist comprises 17 items across three broad categories, namely technological factors (e.g., details of equipment, electrode positioning), safety and noise tests (e.g., reporting of incidents, noise quantification) and methodological factors (e.g., reporting of set-up schematics or the tES-fMRI timing). These critical elements represent suggestions for the minimum required information to ensure reproducibility and to enhance the technical and scientific quality and interpretability of future concurrent fMRI-tDCS studies. Importantly, the checklist is suited to facilitate development of similar guidelines for other imaging modalities.

## 8 Conclusion

To date, there is limited knowledge on how tDCS modulates the complex neural networks supporting higher human cognitive functions in health and disease. Combining neuroimaging technology with tDCS has great potential to reveal the neural mechanisms and predictors underlying behavioral modulation and to identify sources of variability in stimulation response. The present manuscript aimed to discuss the underlying causes of variability in tDCS studies, elaborate on design-related considerations for tDCS-fMRI research, optimization of tDCS and imaging protocols and how to assure high-level experimental control at the level of individual experiments and from a meta-perspective. We also addressed variable tDCS effects across the healthy human lifespan, implications for tDCS studies in age-associated disease, and potential benefits of establishing large-scale, multidisciplinary consortia for more coordinated tDCS research in the future.

We hope that this manuscript will contribute to more coordinated, methodologically sound, transparent and reproducible research in this field, thereby fostering a better understanding of the underlying mechanisms by which tDCS modulates human cognitive functions and ultimately more effective and individually tailored translational and clinical applications of this technique in the future. Ultimately, this will yield information if and how tDCS can modulate human brain functions in a meaningful way.

## Author contributions

MM: Conceptualization, Writing – original draft, Writing – review & editing, Investigation, Methodology, Supervision, Funding acquisition, Resources. AS: Conceptualization, Investigation, Methodology, Supervision, Writing – original draft, Writing – review & editing, Visualization. DA: Writing – review & editing. FB: Writing – review & editing. RF: Writing – review & editing. GH: Writing – review & editing. MAN: Writing – review & editing. S-CL: Writing – review & editing. AT: Writing – review & editing. DT: Writing – review & editing. MA: Writing – original draft, Writing – review & editing. HK: Writing – review & editing. LC: Writing – review & editing. GB: Writing – review & editing. MG: Writing – review & editing. TC: Writing – review & editing. DH: Writing – review & editing. ST: Writing – review & editing. FS: Writing – review & editing. YS: Writing – review & editing. AK: Writing – review & editing. MB: Writing – review & editing. AF: Writing – review & editing, Funding acquisition, Supervision, Writing – original draft. DW: Writing – review & editing. SR: Writing – review & editing. FN: Writing – review & editing.

## Footnotes

^1^ https://soterixmedical.com/research/software/hd-explore

^2^ https://www.humanbrainproject.eu/en/

^3^ https://enigma.ini.usc.edu/

^4^ https://www.youtube.com/@INNN_Network
